# Characterization of intestine-specific TRPM6 knockout C57BL/6 J mice: effects of short-term omeprazole treatment

**DOI:** 10.1007/s00424-024-03017-9

**Published:** 2024-09-13

**Authors:** Anastasia Adella, Lisanne M. M. Gommers, Caro Bos, Pieter A. Leermakers, Jeroen H. F. de Baaij, Joost G. J. Hoenderop

**Affiliations:** Department of Medical BioSciences, Radboud University Medical Center, Nijmegen, The Netherlands

**Keywords:** TRPM6, PPI, PPIH, Hypomagnesemia

## Abstract

The transient receptor potential melastatin type 6 (TRPM6) is a divalent cation channel pivotal for gatekeeping Mg^2+^ balance. Disturbance in Mg^2+^ balance has been associated with the chronic use of proton pump inhibitors (PPIs) such as omeprazole. In this study, we investigated if TRPM6 plays a role in mediating the effects of short-term (4 days) omeprazole treatment on intestinal Mg^2+^ malabsorption using intestine-specific TRPM6 knockout (*Vill1*-TRPM6^−/−^) mice. To do this, forty-eight adult male C57BL/6 J mice (50% TRPM6^fl/fl^ and 50% *Vill1*-TRPM6^−/−^) were characterized, and the distal colon of these mice was subjected to RNA sequencing. Moreover, these mice were exposed to 20 mg/kg bodyweight omeprazole or placebo for 4 days. *Vill1*-TRPM6^−/−^ mice had a significantly lower ^25^Mg^2+^ absorption compared to control TRPM6^fl/fl^ mice, accompanied by lower Mg^2+^ serum levels, and urinary Mg^2+^ excretion. Furthermore, renal *Slc41a3*, *Trpm6*, and *Trpm7* gene expressions were higher in these animals, indicating a compensatory mechanism via the kidney. RNA sequencing of the distal colon revealed a downregulation of the Mn^2+^ transporter *Slc30a10*. However, no changes in Mn^2+^ serum, urine, and feces levels were observed. Moreover, 4 days omeprazole treatment did not affect Mg^2+^ homeostasis as no changes in serum ^25^Mg^2+^ and total Mg^2+^ were seen. In conclusion, we demonstrate here for the first time that *Vill1*-TRPM6^−/−^ mice have a lower Mg^2+^ absorption in the intestines. Moreover, short-term omeprazole treatment does not alter Mg^2+^ absorption in both *Vill1*-TRPM6^−/−^ and TRPM6^fl/fl^ mice. This suggests that TRPM6-mediated Mg^2+^ absorption in the intestines is not affected by short-term PPI administration.

**Supplementary Information:**

The online version contains supplementary material available at 10.1007/s00424-024-03017-9.

## Introduction

The transient receptor potential melastatin type 6 (TRPM6) divalent cation channel is the gatekeeper of intestinal and renal active Mg^2+^ (re)absorption. Loss-of-function mutations in the *TRPM6* gene cause hypomagnesemia with secondary hypocalcemia (HSH; OMIM: 602014) [24, 35]. In mice, TRPM6 activity in extraembryonic tissues is essential for embryonic development, and, consequently, deletion of *Trpm6* results in embryonic lethality [2, 36]. TRPM6 is tightly regulated by a number of upstream modulators. Epidermal growth factor (EGF) and insulin increase TRPM6 translocation to the plasma membrane [20, 28]. At the transcription level, *TRPM6* expression is modulated by EGF and estrogens [8]. pH is also a determinant factor in TRPM6 activity as lower extracellular pH has been shown to increase TRPM6 inward currents [16]. TRPM6 forms a heteromeric complex with its close relative, TRPM7, and fine-tunes Mg^2+^ absorption, further highlighting its importance [5]. In this complex, TRPM6 increases current amplitudes and releases TRPM7 from [Mg-ATP]_i_ inhibition, highlighting its function in alleviating Mg^2+^ transport [2, 3].

Proton pump inhibitors (PPIs) are a class of drugs heavily prescribed worldwide to treat acid peptic diseases (e.g., esophagitis, gastroesophageal reflux disease, and peptic ulcer disease) by targeting the H^+^-K^+^ ATPase found along the stomach parietal cells [18, 22, 23, 25]. Interestingly, chronic PPI users with SNPs in TRPM6 have an increased risk of developing hypomagnesemia [9]. Rodent studies showed that Sprague–Dawley rats that were subjected to prolonged omeprazole treatment had significantly reduced Mg^2+^ absorption accompanied by increased TRPM6 expression [26] and that C57BL/6 J mice that were subjected to 14 days of omeprazole treatment demonstrated increased *Trpm6* gene expression in the colon [13]. In line with this, our previous data showed that 4 weeks of omeprazole treatment resulted in lower serum Mg^2+^ levels in adult mice that were given a low Mg^2+^ diet as a model to stimulate colonic absorption of Mg^2+^ via TRPM6 [6]. Based on these findings, this current study hypothesized that omeprazole decreases Mg^2+^ absorption via TRPM6 in the colon.

This study investigated how short-term omeprazole treatment affects intestinal Mg^2+^ malabsorption and whether TRPM6 is involved using the intestine-specific TRPM6 knockout (*Vill1*-TRPM6^−/−^) mice. To do this, the baseline phenotypes of the *Vill1*-TRPM6^−/−^ mice were characterized and the distal colon of these mice was subjected to RNA sequencing. Furthermore, these mice were exposed to omeprazole or placebo for four consecutive days and several parameters were measured, including intestinal Mg^2+^ absorption using the ^25^Mg^2+^ isotope.

## Results

### Generation of Vill1-TRPM6^−/−^ mice

**Fig. 1:**
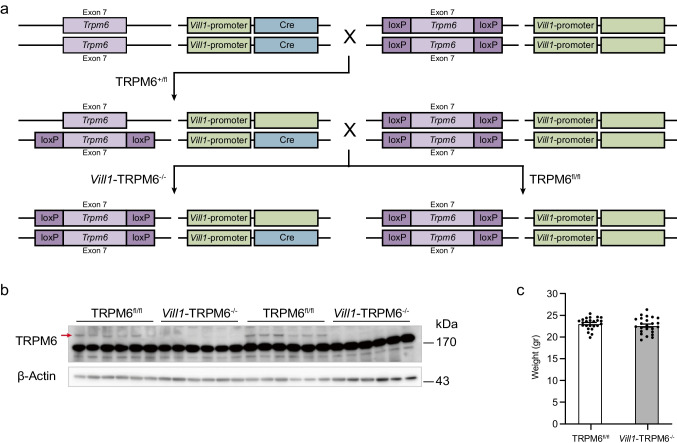
Generation of intestine-specific TRPM6 knockout model. **a** Crossing scheme of *Trpm6* floxed strain with *Villin1-Cre* delete strain to obtain *Vill1*-TRPM6^−/−^ and TRPM6^fl/fl^ littermates. **b** Immunoblot showing expression of TRPM6 (red arrow, expected size 230 kDa) and β-actin as the loading control in the proximal colon of *Vill1*-TRPM6^−/−^ and TRPM6^fl/fl^ mice. **c** Body weight of mice at the beginning of the study, after 24-h in a metabolic cage

*Vill1*-TRPM6^−/−^ mice were generated using the LoxP and *Villin1*-Cre system (Fig. 1a). In short, the *floxed-*TRPM6 (TRPM6^fl/fl^) mice with transgenic mice expressing Cre under the control of the *Villin1* promotor were crossed, resulting in the removal of exon 7 of *Trpm6* by a loxP reporter sequence. From the filial (F) 1 progeny, mice that express the Cre transgene were selected. Back-crossing of these mice with the TRPM6^fl/fl^ strain resulted in pups with TRPM6 deletion under Villin1 promoter (*Vill1*-TRPM6^−/−^). Homozygous TRPM6^fl/fl^ littermates without Cre expression served as the wild-type controls. These F2 progeny mice were used for subsequent studies. The absence of TRPM6 at the protein level from the proximal colon was confirmed by immunoblotting (Fig. 1b). Body weight was not different between genotypes (Fig. 1c).

### Basal characterization of Vill1-TRPM6^−/−^ mice

**Fig. 2:**
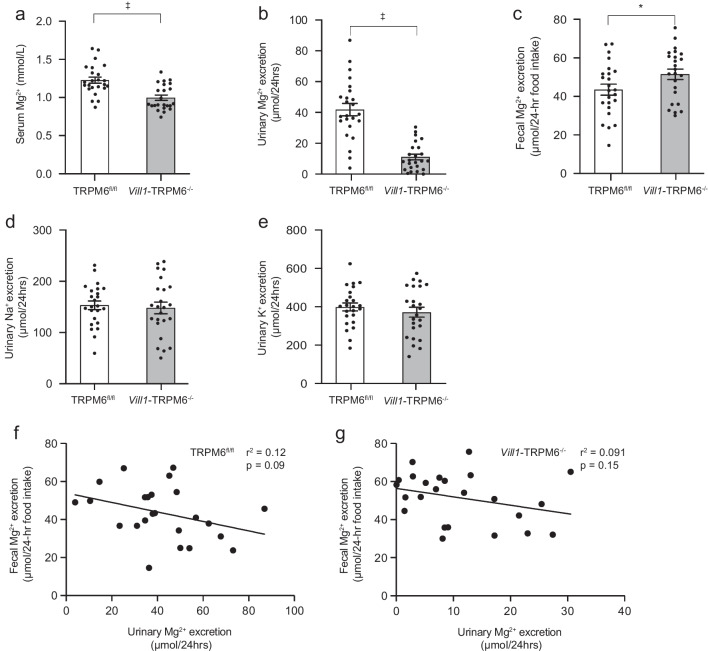
Baseline characterization of TRPM6^fl/fl^ and *Vill1*-TRPM6^−/−^ mice.** a**–**e** Bar graphs depicting serum Mg^2+^ (**a**), 24-h Mg^2+^ urinary excretion (**b**), 24-h Mg^2+^ fecal excretion corrected to 24-h food intake (**c**), urinary Na^+^ excretion (**d**), and urinary K^+^ excretion (**e**). Values are presented as mean ± SEM (*n* = 12). **f**, **g** Correlation analysis between fecal and urinary Mg^2+^ excretion (simple regression analysis) in TRPM6^fl/fl^ (**f**, *y* =  − 0.249x + 53.94) and *Vill1*-TRPM6.^−/−^ (**g**, *y* =  − 0.4422x + 56.4) following 24-h metabolic cage. Significant differences were determined with a one-tailed unpaired *t*-test. ‡ *p* < 0.001

Next, to characterize the baseline phenotypes of *Vill1*-TRPM6^−/−^ and TRPM6^fl/fl^ mice, these mice were kept in individual metabolic cages for 24 h. Serum Mg^2+^ levels were significantly lower in *Vill1*-TRPM6^−/−^ mice compared to TRPM6^fl/fl^ mice (0.99 mmol/L vs. 1.37 mmol/L) (Fig. 2a). *Vill1*-TRPM6^−/−^ mice excreted a much lower amount of urinary Mg^2+^ (41.87 vs. 11.14 µmol/24 h) and higher fecal Mg^2+^ when corrected to 24-h food intake compared to TRPM6^fl/fl^ mice (88.97 vs. 102.14 µmol/24 h food intake) (Fig. 2b, c). Of note, we ran a correlation analysis between urinary and fecal Mg^2+^ excretion and found that higher fecal Mg^2+^ was not associated with lower urinary Mg^2+^ excretion for both genotypes (Fig. 2f, g). No differences in urinary excretion of Na^+^ or K^+^ were detected between genotypes (Fig. 2d, e). Twenty-four-hour food and water intake, as well as 24-h urine and feces production, did not differ between the two genotypes (Figure S1a–d).

### Deletion of Trpm6 from the distal *colon* does not seem to affect the functionality of SLC30A10

**Fig. 3:**
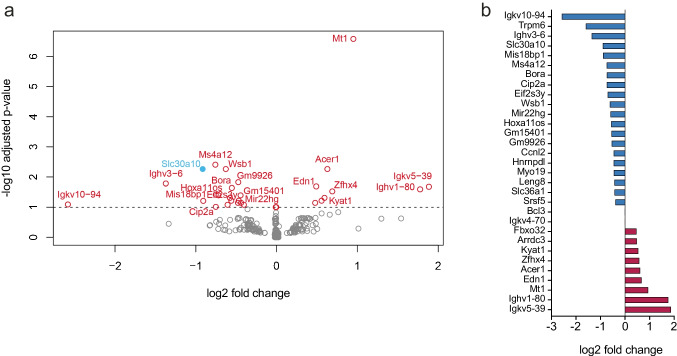
RNA sequencing of the distal colon. **a** Volcano plot of up- or downregulated genes (adjusted *P* < 0.1; in red) in *Vill1*-TRPM6^−/−^ mice treated with placebo compared to TRPM6^fl/fl^ mice, with exclusion of *Trpm6*. Genes with log2 fold change of > 0.5 or <  − 0.5 are labeled. Among these genes, *Slc30a10* was found to be downregulated in *Vill1*-TRPM6^−/−^ mice (blue). **b** Bar graph showing the up- or downregulated genes ranked by log2 fold change in *Vill1*-TRPM6^−/−^ mice

To determine the expression changes in the colon of *Vill1*-TRPM6^−/−^ mice, we compared the transcriptome of the distal colon of *Vill1*-TRPM6^−/−^ to the TRPM6^fl/fl^ mice using RNA sequencing. 31 genes were differentially expressed, including *Trpm6* (Fig. 3a, b, Supplementary Table S1, Supplementary Figure S2). Gene ontology (GO) term and functional annotation analyses did not result in any significantly enriched terms (Supplementary Figure S2d, e). Among these genes, the solute carrier family 30 member 10 (*Slc30a10*) was downregulated in *Vill1*-TRPM6^−/−^ mice (log2 fold change − 0.92). However, no significant differences were observed in Mn^2+^ serum, urine, and feces levels of the *Vill1*-TRPM6^−/−^ mice (Supplementary Figure S3a–c). Serum concentration of Zn^2+^ was decreased in *Vill1*-TRPM6^−/−^ mice, but no differences in fecal and urine excretion (Supplementary Figure S3d–f). In HEK293 cells, cells overexpressing SLC30A10 WT had significantly higher ^25^Mg^2+^ uptake compared to mock and mutant conditions (Supplementary Figure S3g).

### Omeprazole treatment does not change Mg^2+^ levels in TRPM6^fl/fl^ and Vill1-TRPM6^−/−^ mice

**Fig. 4:**
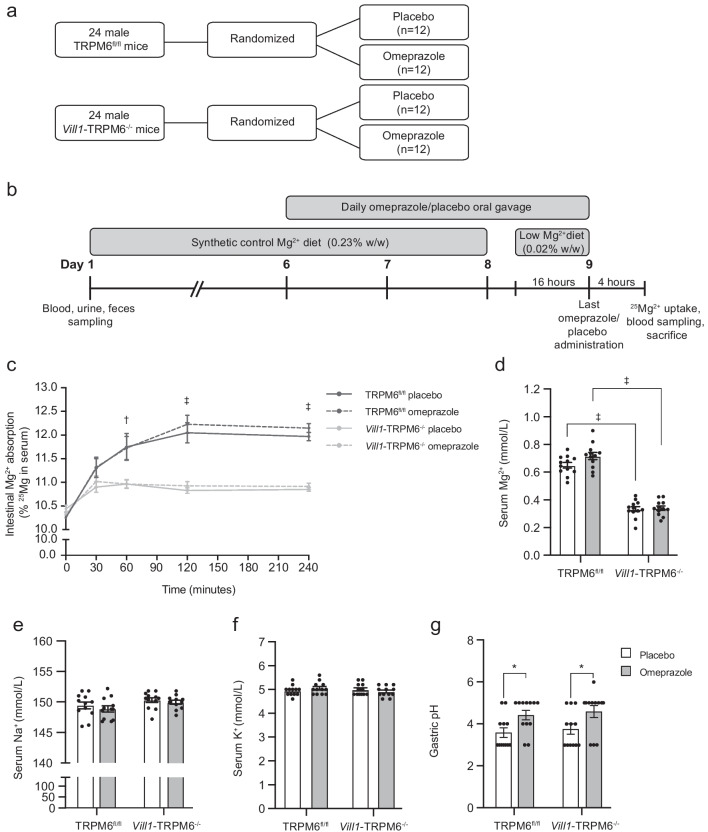
Effects of omeprazole treatment in TRPM6^fl/fl^ and *Vill1*-TRPM6^−/−^ mice. **a** CONSORT-like scheme of the study design. **b** Timeline of the study. **c** Relative ^25^Mg^2+^ serum levels of TRPM6^fl/fl^ (black lines) and *Vill1*-TRPM6^−/−  ^(light grey lines) mice, after placebo (solid continuous lines) or omeprazole treatment (dashed lines). Values are presented as mean ± SEM (*n* = 12). Significant differences between genotypes in each time point were determined with Two-way ANOVA followed by Šídák post-hoc test. **d**–**g** Bar graphs depicting serum Mg^2+^ (**d**), serum Na^+^ (**e**), and serum K^+^ (**f**) levels in TRPM6^fl/fl^ and *Vill1*-TRPM6.^−/−^ mice treated with placebo (white bars) or omeprazole (grey bars). Values are presented as mean ± SEM (*n* = 12). Significant differences (*p* < 0.05) were determined with Two-way ANOVA followed by Tukey post-hoc test. **g** Measurement of stomach pH presented as mean ± SEM. The statistical test used was the Mann–Whitney test. *p < 0.05, †*p* < 0.01, ‡*p* < 0.001

To evaluate if PPI-induced hypomagnesemia is mediated by TRPM6 in the colon, TRPM6^fl/fl^ and *Vill1*-TRPM6^−/−^ mice were treated with omeprazole or placebo for four consecutive days following the baseline metabolic cage measurement (Fig. 4a, b). Serum Mg^2+^ levels do not solely reflect intestinal Mg^2+^ uptake but also depend on regulation by the kidney and the bones. Therefore, we performed an intestinal ^25^Mg^2+^ uptake assay 4 h following the last omeprazole administration to isolate intestinal Mg^2+^ absorption from systemic effects. Serum ^25^Mg^2+^ levels are overall significantly lower in Vill1-TRPM6^−/−^ mice compared to TRPM6^fl/fl^ mice, independent of drug treatment (Fig. 4c). Next, we performed follow-up two-way ANOVA followed by Šídák multiple comparison tests on placebo-treated animals. Mg^2+^ intestinal absorption is significantly lower in *Vill1*-TRPM6^−/−^ mice after 60, 120, and 240 min of ^25^Mg^2+^ administration (Fig. 4c). This trend was also reflected in the serum Mg^2+^ levels, which remained significantly reduced in *Vill1*-TRPM6^−/−^ mice compared to TRPM6^fl/fl^ mice (Fig. 4d). No effects of placebo- and omeprazole treatment were seen (Fig. 4c, d). Serum Na^+^ and K^+^ levels remained similar for all genotypes and treatments (Fig. 4e, f). Lastly, as a control for the efficacy of the omeprazole treatment, a higher gastric pH was measured in omeprazole-treated groups (Fig. 4g).

### The absence of TRPM6 from the intestine affects gene expressions of renal Mg^2+^ transporters

**Fig. 5:**
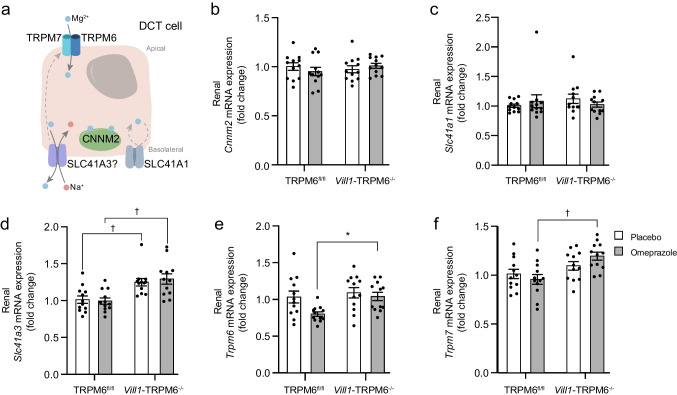
Lack of TRPM6 affects the expression of renal Mg^2+^ transporters. **a** Illustration of the putative transporters involved in Mg^2+^ transport in the DCT. **b**–**f** The renal mRNA expression levels of *Cnnm2* (**b**)*, Slc41a1* (**c**)*, Slc41a3* (**d**)*, Trpm6* (**e**)*, Trpm7* (**f**) in TRPM6^fl/fl^ and *Vill1*-TRPM6^−/−^ mice treated with placebo (white bars) or omeprazole (grey bars). Values are presented as fold change relative to the placebo-treated TRPM6.^fl/fl^ mice group in mean ± SEM (*n* = 12). Significant differences (*p* < 0.05) were determined with Two-way ANOVA followed by Tukey post-hoc test. *p < 0.05, †*p* < 0.01

To examine compensatory mechanisms for the lack of intestinal *Trpm6* and the lower urinary Mg^2+^ excretion in *Vill1*-TRPM6^−/−^ mice, gene expression of various Mg^2+^ transporters in the kidney was quantified (Fig. 5a–f). Renal *Cnnm2* and *Slc41a1* gene expressions were not changed in *Vill1*-TRPM6^−/−^ mice compared to TRPM6^fl/fl^ mice regardless of treatment (Fig. 5b, c). However, *Vill1*-TRPM6^−/−^ mice had higher *Slc41a3* gene expression compared to TRPM6^fl/fl^ (Fig. 5d). Gene expression of *Trpm6* and its close homolog, *Trpm7*, was higher in omeprazole-treated *Vill1*-TRPM6^−/−^ mice compared to omeprazole-treated TRPM6^fl/fl^ mice, but not among the placebo-treated groups (Fig. 5e, f).

## Discussion

In this study, we examined if intestinal TRPM6 mediates Mg^2+^ malabsorption after short-term omeprazole treatment. Our basal characterization of these mice showed that mice lacking intestinal *Trpm6* had lower Mg^2+^ serum levels compared to TRPM6^*fl/fl*^ mice, and importantly, were able to absorb significantly less Mg^2+^ in the intestines as demonstrated by a percentage of ^25^Mg^2+^ in the serum. Four-day omeprazole administration did not affect intestinal Mg^2+^ absorption in both *Vill1*-TRPM6^−/−^ and TRPM6^*fl/fl*^ mice, suggesting that PPI does not affect TRPM6-mediated Mg^2+^ absorption in the short term.

We demonstrated here for the first time that *Vill1*-TRPM6^−/−^ mice have a decreased intestinal Mg^2+^ absorption compared to TRPM6^fl/fl^ mice. As a consequence, *Vill1*-TRPM6^−/−^ mice displayed lower serum Mg^2+^ levels and urinary Mg^2+^ excretion. This was accompanied by a slight increase in renal *Slc41a3, Trpm6,* and *Trpm7* gene expressions, indicating renal compensation. Additionally, 24-h fecal Mg^2+^ excretion was significantly higher in *Vill1*-TRPM6^−/−^ mice than in TRPM6^fl/fl^ mice. These findings are in line with a previous study by Chubanov et al. [2]*.* where global *Trpm6* deficient mice under *Sox2*-Cre expression have a higher fecal Mg^2+^ excretion, lower urinary Mg^2+^ excretion, and bone magnesium content. Moreover, intestine-specific *Trpm6* deficient mice under *Villin1-*Cre displayed hypomagnesemia, lower urinary Mg^2+^ excretion, and bone magnesium content [2]. Altogether, these results support the notion that TRPM6 is important in the intestines for Mg^2+^ absorption.

To find compensatory mechanisms for the loss of TRPM6 in the intestinal tract, the distal colon of these animals was subjected to RNA sequencing. Among others, *Slc30a10* was found to be downregulated in mice lacking intestinal *Trpm6*. SLC30A10 belongs to a cation diffusion facilitator superfamily called SLC30 [11]. SLC30A10 has been established as an efflux transporter with a strong affinity to Mn^2+^ that is expressed in the liver, brain, and gastrointestinal tract [19, 21, 27]. It protects against Mn^2+^ toxicity in cells and mutations in *Slc30a10* result in hypermagnesemia with dystonia 1 (HMNDYT1; OMIM: 613,280) [21, 32, 33]. Due to property similarities between Mg^2+^ and Mn^2+^, we hypothesized that SLC30A10 also has an affinity for Mg^2+^-extrusion. Indeed, we confirmed a limited Mg^2+^ transport capacity for SLC30A10 with ^25^Mg^2+^ uptake assays in HEK293 cells. This suggests that suppressing *Slc30a10* expression works compensatory to maintain systemic Mg^2+^ levels. Of note, although SLC30A10 is capable of transporting Mg^2+^ in our in vitro experiments, the limited transport capacity suggests that the role of SLC30A10 in normal physiology will be minor. Indeed, Mn^2+^ serum, urine, and feces levels were unchanged in *Vill1*-TRPM6^−/−^ mice. This potential role of SLC30A10 in Mg^2+^ transport should be addressed in future studies. On a side note, we observed a lower Zn^2+^ serum concentration in transgenic animals. This might be due to the fact that TRPM6 has a high permeability to Zn^2+^ [31].

Several clinical and experimental studies have proposed that TRPM6 activity is affected by PPI use, resulting in PPI-induced hypomagnesemia [7]. One of these hypotheses suggests that PPI-induced luminal pH change affects TRPM6 activity [7]. To gain more molecular insights into this mechanism, we treated *Vill1*-TRPM6^−/−^ and TRPM6^*fl/fl*^ mice with omeprazole for four consecutive days to detect early PPI-induced changes in Mg^2+^ absorption, even before plasma Mg^2+^ levels would be reduced as this would activate compensatory mechanisms such as increased TRPM6 expression [13, 34]. However, we found no difference in Mg^2+^ levels in serum, urine, and feces, as well as intestinal Mg^2+^ absorption. This is in line with our preceding studies in which C57BL/6 J mice treated with omeprazole had a slight to no changes in serum, urine, and feces Mg^2+^ levels compared to vehicle [6, 10, 13]. Altogether, these results question whether C57BL/6 J mice are a good model to study PPI-induced hypomagnesemia.

There are some limitations to our study. First, the effects of omeprazole were assessed after only a short period of treatment. Previously, we and other groups have shown that longer treatment (> 2 weeks) of omeprazole increased TRPM6 expression [13, 26, 30]. Moreover, long-term omeprazole treatment in Sprague–Dawley rats (12–24 weeks) lowered plasma and urinary Mg^2+^ [26]. Therefore, we cannot exclude that a longer duration of omeprazole treatment would induce differences in Mg^2+^ absorption. Indeed, although the precise duration of PPI use, until hypomagnesemia symptoms appear, is not clear, PPI users have been reported to have a higher risk of developing PPI-induced hypomagnesemia only after a longer use (> 6 months) [12]. Combined with our study results, this suggests that the effects of omeprazole on Mg^2+^ levels are dependent on chronic exposure. Thus, future studies should be performed with prolonged omeprazole treatment (> 2 weeks), to characterize the role of intestinal TRPM6 in chronic adaptation to omeprazole. Second, it is known that low luminal Mg^2+^ stimulates more distal absorption of Mg^2+^ compared to the proximal parts of the intestines, while high Mg^2+^ favors proximal absorption [14]. However, in addition to the colon, *Trpm6* is also expressed in more proximal parts of the intestines, albeit significantly lower than in the colon [14]. Therefore, it is possible that the lower absorption observed at earlier time points (i.e., 30 and 60 min) is due to a proximal defect. In this study, we did not look into colon-specific Mg^2+^ transport. A segment-specific study such as using Ussing chamber could be performed in future studies to identify the effects of TRPM6 knockout in the colon. Lastly, only male mice were used in this study. TRPM6 is known to be activated by estrogens [8], and therefore, estrogens might affect the functionality of TRPM6 under omeprazole treatment. In the future, it would be important to investigate how the different sexes respond to PPI treatment.

In conclusion, this study demonstrates that TRPM6 is essential to maintain intestinal Mg^2+^ absorption. Furthermore, we established that this intestinal Mg^2+^ absorption by TRPM6 is not affected by short-term omeprazole administration.

## Materials and methods

### Animal genetic background

Ethical approval was obtained from the ethics board of Radboud University (DEC 2017–0024) and the Dutch Central Commission for Animal Experiments (AVD1030020173224). Power calculation to determine the appropriate sample size per group (*n* = 12) was performed prior to the study. Floxed *Trpm6* (B6NCrl;B6N-A^tm1Brd^ Trpm6^tm1a(KOMP)Wtsi^/CipheOrl; EM:10,341, EMMA/Infrafrontier, France) mice, hereby termed TRPM6^fl/fl^, were purchased from INFRARONTIER/EMMA (www.infrafrontier.eu, PMID: 25,414,328) [4]. *Villin1*-Cre delete strain (B6N.Cg-Tg(Vil1-cre)997Gum/J, stock #018963, The Jackson Laboratory, ME USA) was obtained from The Jackson Laboratory. Genotyping of the floxed *Trpm6* allele was performed using primers available in Table 1.

**Table 1:** List of primer sequences used in this study

Name	Species	Forward primer (5′-3′)	Reverse primer (5′-3′)	Use
*Trpm6* floxed	Mouse	CCTCTCTCTGCTCCTCAGGGTTCC	CAACAATACCCACACATATCCTGCCC	Genotyping
*Cre*	Mouse	GCCTGCATTACCGGTCGATGC	CGACCGGCAAACGGACAGAAGC	RT-qPCR
*Cnnm2*	Mouse	GTCTCGCACCTTTGTTGTCA	GTCGCTCCGACTGAGAGAAT	RT-qPCR
*Slc41a1*	Mouse	TCCCTGATGGCCACTTTAGC	GATCATACCCAGGACCAAGGAG	RT-qPCR
*Slc41a3*	Mouse	TGAAGGGAAACCTGGAAATG	GGTTGCTGCTGATGATTTTG	RT-qPCR
*Trpm6*	Mouse	GGTTGCTGCTGATGATTTTG	GGTTGCTGCTGATGATTTTG	RT-qPCR
*Trpm7*	Mouse	GGTTCCTCCTGTGGTGCCTT	CCCCATGTCGTCTCTGTCGT	RT-qPCR
SLC30A10 WT	Human	CCTTCTTCGTGGCGGAGCTGGTCTCCGGCTAC	GTAGCCGGAGACCAGCTCCGCCACGAAGAAGG	Mutagenesis

### Animal studies

In total 48 adult (8–10 weeks old) males (50% TRPM6^fl/fl^, 50% *Vill1*-TRPM6^−/−^) were included in this study. After acclimatization for 2 weeks to synthetic chow with regular composition (calcium 0.92%, phosphorus 0.65%, sodium 0.2%, magnesium 0.23%, potassium 0.97%, w/w) (E15000-04, Ssniff Spezialdiäten, GmbH, Germany), mice were housed individually in metabolic cages for 24 h to collect urine and feces and monitor food and water intake. Blood was drawn via submandibular vein puncture and collected in microvette tubes (Sarstedt, Nümbrecht, Germany). Blood was centrifuged at 3500 × *g* for 5 min after coagulation to obtain serum.

Six days after the metabolic cages, 50% of mice from respective genotypes (*n* = 12 per group) were subjected to daily administration with either omeprazole (20 mg/kg bodyweight) or placebo (0.5% (w/v) methylcellulose and 0.2% (w/v) NaHCO_3_, adjusted with NaOH to pH 9.0) via oral gavage for 4 days (Fig. 4A). The night before sacrifice, the feed for all groups was switched from normal Mg^2+^ to a low (same composition as regular feed but with 0.02% magnesium, w/w) Mg^2+^ diet (S9074-E1007 EF E15000, Ssniff Spezialdiäten, GmbH, Germany) and placed on wire-mesh raised floors to prevent coprophagia.

On the last day, approximately 4 h after the last vehicle or omeprazole treatment, mice were given the stable isotope ^25^Mg^2+^ (MgO, CortectNet, Voisins-Le-Bretonneux, France) via oral gavage, as described before [14]. At time point-0, 15 µL/g bodyweight of 44 mM ^25^Mg^2+^, 125 mM NaCl, 17 mM Tris–HCl pH 7.5, and 1.8 g/L fructose was administered to the animals via oral gavage. Blood samples were taken by cutting off the end of the tail of the mice and subsequent collection in glass capillaries (128,137, Praxisdienst). Blood samples were taken at 0-, 30-, 60-, 120-, 180-, and 240-min after administration of the ^25^Mg^2+^.

At the end of the study, mice were anesthetized with 4% (v/v) isoflurane and sacrificed via exsanguination via orbital sinus bleeding and subsequent cervical dislocation. Blood and organs were collected for further analysis.

### Electrolyte measurements

Serum and urine Na^+^ and K^+^ measurements were performed by the clinical laboratory of Radboudumc using an automated analysis system (Abbott Diagnostics, The Netherlands). To analyze Mg^2+^, Mn^2+^, and Zn^2+^, serum, urine, and feces samples were diluted in nitric acid (> 65%, Sigma, The Netherlands) and milliQ. These samples were then sent for ICP-MS analysis (Faculty of Science, Radboud University, Nijmegen, The Netherlands). The percentage of ^25^Mg^2+^ was calculated by making a ratio between ^25^Mg^2+^ and total Mg^2+^ (^24^Mg^2+^  + ^25^Mg^2+^  + ^26^Mg^2+^). To measure ^24^Mg^2+^, ^55^Mn^2+^, and ^66^Zn^2+^ serum, urine, and feces samples were diluted in milliQ and nitric acid, and subjected to ICP-MS (General Instrumentation, Faculty of Science, Radboud University).

### RNA isolation and quantitative real-time PCR

At the end of the study, total RNA was extracted from the kidney, proximal, and distal colon using Trizol Reagent (Invitrogen, Bleiswijk, The Netherlands) according to the manufacturer’s protocol. Next, 1 µg of isolated RNA was treated with DNase (Promega, Fitchburg, WI, USA) and reverse transcribed with Moloney Murine Leukemia Virus Reverse Transcriptase (Invitrogen, Bleiswijk, The Netherlands). Obtained samples were stored at − 20 °C.

Expression of genes was quantified using SYBR green (Bio-Rad, Hercules, CA, USA) on a CFX96 Real-Time PCR Detection System (Bio-Rad) and normalized for *Gapdh*. To calculate relative gene expression, 2^−ΔΔCt^ method was used. Values are displayed as fold changes to the control group. All sequences of primers used are listed in Table 1.

### RNA sequencing

Total RNA was isolated from the distal colon of the mice as described above. Next, RNA-Seq libraries were prepared from total RNA using the KAPA RNA HyperPrep Kit with RiboErase (KAPA Biosystems, Wilmington, MA, USA). Briefly, oligo hybridization and rRNA depletion, rRNA depletion cleanup, DNase digestion, DNase digestion cleanup, and RNA elution were performed according to the manufacturer’s protocol. Fragmentation and priming were performed at 94 °C for 6 min. Synthesis of the first- and second-strand, and A-tailing were performed according to the protocol. The adaptor was ligated using NextFlex DNA barcodes (1.5 mM stock; Bio Scientific, Austin, TX). Further, the first and second post-ligation cleanup was performed according to the protocol. To amplify the library, 11 PCR cycles were performed and further cleaned up using a 0.8 × followed by 1.0 × bead-based cleanup. Library size was determined using the High Sensitivity DNA bioanalyzer kit, and the library concentration was measured using the dsDNA High Sensitivity Assay (DeNovix, Wilmington, DE, USA). Paired-end sequencing reads of 50 bp were generated using an Illumina NextSeq 2000.

### RNA-Seq data analysis

RNA-Seq data were analyzed as previously described [29] using the seq2science pipeline (https://vanheeringen-lab.github.io/seq2science/content/workflows/rna_seq.html). In short, reads were aligned to the mm10 reference transcript assembly from UCSC. Next, reads were filtered using SAMtools (RRID: RRID:SCR_002105), and quality score lower than 20, and PCR duplicates were removed [15]. Reads per gene were counted with the htseq-count script from the Hisat2 software suite using the GTF file corresponding to the transcript assembly. Read counts were further analyzed with DESeq2 (RRID: SCR_002285) [17]. RNA-Seq data were deposited in the National Center for Biotechnology Information Gene Expression Omnibus (GEO) database (Accession No. GSE243832).

### DNA constructs

pCMV-FLAG10-SLC30A10-D248A (Addgene plasmid #82,346;) and pCMV-FLAG10-SLC30A10-E25A (Addgene plasmid #82,345) constructs were gifts from Somshuvra Mukhopadhyay [37]. To obtain wild-type (WT) SLC30A10 construct, the WT sequence was inserted in the SLC30A10 E25A construct using the QuikChange site-directed mutagenesis kit (Stratagene, La Jolla, CA, USA) according to the manufacturer’s protocol. To obtain an empty pCMV-FLAG10 construct (mock), the SLC30A10 gene was removed from the SLC30A10 E25A construct by digestion with EcoRI-HF (New England Biolabs) and subsequent DNA purification. All constructs were verified by sequencing analysis. Primer sequences for mutagenesis PCR are shown in Table 1.

### Cell culture

HEK293 cells were grown in Dulbecco’s modified Eagle’s medium (DMEM, Lonza) containing 10% (v/v) fetal calf serum (VWR International), 2 mM L-glutamine (Sigma-Aldrich), and 10 µg/mL nonessential amino acids (Sigma-Aldrich) at 37 °C and 5% (v/v) CO_2_. To transfect the cells with mock, WT, or mutant SLC30A10, Lipofectamine 2000 (Invitrogen) was used at 1:2 DNA:Lipofectamine ratio.

### ^25^Mg^2+^ uptake assay

HEK293 cells were seeded as previously described [1]. In short, HEK293 cells were transfected with mock, WT, or mutant SLC30A10 as described above. Sixteen hours later, the transfected cells were re-seeded in Poly-L-Lysine coated 12-well plates. Twenty-four hours following re-seeding, cells were washed 1 × with phosphate-buffered saline (PBS) and incubated with Mg^2+^ free uptake buffer (125 mM NaCl, 5 mM KCl, 0.5 mM CaCl_2_, 0.5 mM Na_2_HPO_4_, 0.5 mM Na_2_SO_4_, 15 mM HEPES, adjusted to pH 7.5 using NaOH) supplemented with 1 mM ^25^Mg^2+^ for 15 min. Afterward, cells were washed 3 × with ice-cold PBS and lysed in nitric acid before being sent for ICP-MS analysis (General Instrumentation, Faculty of Science, Radboud University).

### SDS-PAGE and Western blot analysis

Proximal colon and kidney tissues were homogenized in triton lysis buffer (1 mM EDTA, 1 mM EGTA, 10 mM C_3_H_7_Na_2_O_6_P, 50 mM NaF, 10 mM Na_4_P_2_O_7_, 150 mM NaCl, 270 mM sucrose, 50 mM Tris–HCl pH 7.5, 1% [v/v] Triton X-100) supplemented with protease and phosphatase inhibitors (1.46 nM Pepstatin, 10.5 nM Leupeptin, 1 mM PMSF, 0.154 nM Aprotinin, 1 mM Na_3_VO_4_) using Ultra-Turrax-T25 followed by Dounce homogenizer. Cells were homogenized by scraping in triton lysis buffer. Samples were then clarified by centrifugation at 1000 × *g* for 10 min at 4 °C and supernatants were transferred to new tubes. Protein concentration was measured using the Pierce™ BCA Protein Assay Kit (Thermo Scientific). Next, samples were denatured in 5 × Laemmli buffer containing 1 mM dithioterol (DTT) at 37 °C for 30 min.

Ten to 15 µg of lysate was run through SDS-PAGE and transferred to a polyvinylidene fluoride membrane and blocked with 5% (w/v) non-fat dry milk in Tris-buffered saline (TBS) with 0.1% (v/v) Tween-20 (TBS-T; Sigma-Aldrich) for 1 h at RT. Subsequently, membranes were incubated in primary antibody diluted in 1% (w/v) non-fat dry milk in TBS-T overnight at 4 °C. The next day, membranes were washed 3 × with TBS-T and incubated with peroxidase (PO) conjugated secondary antibodies (Roche, Mannheim, Germany) for 1 h at RT. This was followed by 3 × TBS-T washes and 1 × TBS wash. Lastly, proteins of interest were visualized by incubating membranes with SuperSignal West Pico Chemiluminescent Substrate (Thermo Scientific) or SuperSignal West Femto Maximum Sensitivity Substrate (Thermo Scientific) using the ImageQuant™ LAS 4000 (General Electric). Primary antibodies used are against TRPM6 used at 1:800 (#ACC-046, Alomone Labs) and B-actin used at 1:10,000 (#A5441-0.2 mL, Sigma). Secondary antibodies used are PO conjugated against IgG mouse (#145–515-035, Brunswig) and IgG rabbit (#A4914, Sigma) both used at 1:10,000.

### Statistical analysis

All results are depicted as individual values and mean ± SEM. When there were only two experimental groups, a one-tailed unpaired *t*-test was used. When there were more than two experimental groups, with two variables, two-way ANOVA followed by a post-hoc test was used. In experiments with more than two groups and two variables, three-way ANOVA was used. The test used in each experiment is indicated in each figure legend. Statistical significance was described at *p* < 0.05, depicted as symbols *p < 0.05, †*p* < 0.01, ‡*p* < 0.001. All graphs and statistical tests were run in GraphPad Prism version 10.0.2 (171) for macOS, GraphPad Software, Boston, MA, USA unless stated otherwise.

## Supplementary Information

Below is the link to the electronic supplementary material.

Supplementary file1 Supplementary Table S1. List of differentially expressed genes in distal colon of *Vill1*-TRPM6^-/-^ and TRPM6^fl/fl ^ mice. Adjusted p < 0.1. Supplementary Figure S1. Metabolic parameters of *Vill1*-TRPM6^-/-^ and TRPM6^fl/fl^ mice. (a-d) Bar graphs depicting 24-hour feed intake (a), 24-hour water intake (b), 24-hour urine production (c), 24-hour faeces production (d). Values are presented as mean ± SEM (n=12) and statistical significance (p < 0.05) was determined with one-tailed unpaired T-test. (e-f) Uncropped immunoblots of TRPM6 (e) and B-actin (f), both pointed by red arrows, in the proximal colon. Supplementary Figure S2. Quality control of RNA-sequencing analysis. (a) Principal component analysis (PCA) based on normalized counts of six independent RNA samples from the distal colon of three TRPM6^fl/fl^ (WT1-3) and three *Vill1*-TRPM6^-/-^ (KO1-3) mice generated with DESeq2 R-package. (b) Heatmap of Pearson correlation clustering of the six samples based on variance stabilizing transformed counts generated with DESeq2 R-package. (c) Heatmap showing pairwise correlations of samples based on distribution of sequence reads. (d-e) Gene Ontology (GO) term enrichment analysis (d) and functional annotation (e) of differentially expressed genes. Supplementary Figure S3. Functionality of SLC30A10. Mn^2+^ levels in serum (a), 24-hour urine (b), and 24-hour feces corrected to 24-hr food intake (c) from TRPM6^fl/fl^ and *Vill1*-TRPM6^-/-^ mice. (d-f) Zn^2+^ levels in serum (d), 24-hour urine (e), and 24-hour feces corrected to 24-hour food intake (f). ^25^Mg^2+^ uptake by HEK293 cells overexpressing mock, SLC30A10 WT, SLC30A10 D248A, and SLC30A10 (g). Values are presented as mean ± SEM (a-f: n=12; g: N=3 independent experiments). Significant differences (p < 0.05) were determined with one-tailed unpaired T-test (a-f) and One-way ANOVA followed by Tukey post-hoc test (g). * p < 0.05, † p < 0.01. (DOCX 1287 KB)

## Data Availability

The data that support the findings of this study are available from the corresponding author upon reasonable request.

## References

[1] Arjona FJ, de Baaij JH, Schlingmann KP, Lameris AL, van Wijk E, Flik G, Regele S, Korenke GC, Neophytou B, Rust S, Reintjes N, Konrad M, Bindels RJ, Hoenderop JG (2014) CNNM2 mutations cause impaired brain development and seizures in patients with hypomagnesemia. PLoS Genet 10:e1004267. 10.1371/journal.pgen.1004267 24699222 10.1371/journal.pgen.1004267 PMC3974678

[2] Chubanov V, Ferioli S, Wisnowsky A, Simmons DG, Leitzinger C, Einer C, Jonas W, Shymkiv Y, Bartsch H, Braun A, Akdogan B, Mittermeier L, Sytik L, Torben F, Jurinovic V, van der Vorst EP, Weber C, Yildirim OA, Sotlar K, Schurmann A, Zierler S, Zischka H, Ryazanov AG, Gudermann T (2016) Epithelial magnesium transport by TRPM6 is essential for prenatal development and adult survival. Elife 5. 10.7554/eLife.20914 10.7554/eLife.20914 PMC5218537 27991852

[3] Chubanov V, Waldegger S, Mederos y Schnitzler M, Vitzthum H, Sassen MC, Seyberth HW, Konrad M, Gudermann T (2004) Disruption of TRPM6/TRPM7 complex formation by a mutation in the TRPM6 gene causes hypomagnesemia with secondary hypocalcemia. Proc Natl Acad Sci U S A 101:2894–2899. 10.1073/pnas.0305252101 14976260 10.1073/pnas.0305252101 PMC365716

[4] Consortium I (2015) INFRAFRONTIER–providing mutant mouse resources as research tools for the international scientific community. Nucleic Acids Res 43:D1171-1175. 10.1093/nar/gku1193 25414328 10.1093/nar/gku1193 PMC4383977

[5] de Baaij JH, Hoenderop JG, Bindels RJ (2015) Magnesium in man: implications for health and disease. Physiol Rev 95:1–46. 10.1152/physrev.00012.2014 25540137 10.1152/physrev.00012.2014

[6] Gommers LMM, Ederveen THA, van der Wijst J, Overmars-Bos C, Kortman GAM, Boekhorst J, Bindels RJM, de Baaij JHF, Hoenderop JGJ (2019) Low gut microbiota diversity and dietary magnesium intake are associated with the development of PPI-induced hypomagnesemia. FASEB J 33:11235–11246. 10.1096/fj.201900839R 31299175 10.1096/fj.201900839R

[7] Gommers LMM, Hoenderop JGJ, de Baaij JHF (2022) Mechanisms of proton pump inhibitor-induced hypomagnesemia. Acta Physiol (Oxf) 235:e13846. 10.1111/apha.13846 35652564 10.1111/apha.13846 PMC9539870

[8] Groenestege WM, Hoenderop JG, van den Heuvel L, Knoers N, Bindels RJ (2006) The epithelial Mg2+ channel transient receptor potential melastatin 6 is regulated by dietary Mg2+ content and estrogens. J Am Soc Nephrol 17:1035–1043. 10.1681/ASN.2005070700 16524949 10.1681/ASN.2005070700

[9] Hess MW, de Baaij JH, Broekman MM, Bisseling TM, Haarhuis BJ, Tan AC, Te Morsche RH, Hoenderop JG, Bindels RJ, Drenth JP (2017) Common single nucleotide polymorphisms in transient receptor potential melastatin type 6 increase the risk for proton pump inhibitor-induced hypomagnesemia: a case-control study. Pharmacogenet Genomics 27:83–88. 10.1097/FPC.0000000000000259 27926584 10.1097/FPC.0000000000000259

[10] Hess MW, de Baaij JH, Gommers LM, Hoenderop JG, Bindels RJ (2015) Dietary Inulin fibers prevent proton-pump inhibitor (PPI)-induced hypocalcemia in mice. PLoS ONE 10:e0138881. 10.1371/journal.pone.0138881 26397986 10.1371/journal.pone.0138881 PMC4580428

[11] Huang L, Tepaamorndech S (2013) The SLC30 family of zinc transporters - a review of current understanding of their biological and pathophysiological roles. Mol Aspects Med 34:548–560. 10.1016/j.mam.2012.05.008 23506888 10.1016/j.mam.2012.05.008

[12] Kieboom BC, Kiefte-de Jong JC, Eijgelsheim M, Franco OH, Kuipers EJ, Hofman A, Zietse R, Stricker BH, Hoorn EJ (2015) Proton pump inhibitors and hypomagnesemia in the general population: a population-based cohort study. Am J Kidney Dis 66:775–782. 10.1053/j.ajkd.2015.05.012 26123862 10.1053/j.ajkd.2015.05.012

[13] Lameris AL, Hess MW, van Kruijsbergen I, Hoenderop JG, Bindels RJ (2013) Omeprazole enhances the colonic expression of the Mg(2+) transporter TRPM6. Pflugers Arch 465:1613–1620. 10.1007/s00424-013-1306-0 23756852 10.1007/s00424-013-1306-0

[14] Lameris AL, Nevalainen PI, Reijnen D, Simons E, Eygensteyn J, Monnens L, Bindels RJ, Hoenderop JG (2015) Segmental transport of Ca(2)(+) and Mg(2)(+) along the gastrointestinal tract. Am J Physiol Gastrointest Liver Physiol 308:G206-216. 10.1152/ajpgi.00093.2014 25477372 10.1152/ajpgi.00093.2014

[15] Li H, Handsaker B, Wysoker A, Fennell T, Ruan J, Homer N, Marth G, Abecasis G, Durbin R, Genome Project Data Processing S (2009) The sequence alignment/map format and SAMtools. Bioinformatics 25:2078–2079. 10.1093/bioinformatics/btp352 19505943 10.1093/bioinformatics/btp352 PMC2723002

[16] Li M, Jiang J, Yue L (2006) Functional characterization of homo- and heteromeric channel kinases TRPM6 and TRPM7. J Gen Physiol 127:525–537. 10.1085/jgp.200609502 16636202 10.1085/jgp.200609502 PMC2151519

[17] Love MI, Huber W, Anders S (2014) Moderated estimation of fold change and dispersion for RNA-seq data with DESeq2. Genome Biol 15:550. 10.1186/s13059-014-0550-8 25516281 10.1186/s13059-014-0550-8 PMC4302049

[18] Mejia A, Kraft WK (2009) Acid peptic diseases: pharmacological approach to treatment. Expert Rev Clin Pharmacol 2:295–314. 10.1586/ecp.09.8 21822447 10.1586/ecp.09.8 PMC3149864

[19] Mercadante CJ, Prajapati M, Conboy HL, Dash ME, Herrera C, Pettiglio MA, Cintron-Rivera L, Salesky MA, Rao DB, Bartnikas TB (2019) Manganese transporter Slc30a10 controls physiological manganese excretion and toxicity. J Clin Invest 129:5442–5461. 10.1172/JCI129710 31527311 10.1172/JCI129710 PMC6877324

[20] Nair AV, Hocher B, Verkaart S, van Zeeland F, Pfab T, Slowinski T, Chen YP, Schlingmann KP, Schaller A, Gallati S, Bindels RJ, Konrad M, Hoenderop JG (2012) Loss of insulin-induced activation of TRPM6 magnesium channels results in impaired glucose tolerance during pregnancy. Proc Natl Acad Sci U S A 109:11324–11329. 10.1073/pnas.1113811109 22733750 10.1073/pnas.1113811109 PMC3396482

[21] Quadri M, Federico A, Zhao T, Breedveld GJ, Battisti C, Delnooz C, Severijnen LA, Di Toro ML, Mignarri A, Monti L, Sanna A, Lu P, Punzo F, Cossu G, Willemsen R, Rasi F, Oostra BA, van de Warrenburg BP, Bonifati V (2012) Mutations in SLC30A10 cause parkinsonism and dystonia with hypermanganesemia, polycythemia, and chronic liver disease. Am J Hum Genet 90:467–477. 10.1016/j.ajhg.2012.01.017 22341971 10.1016/j.ajhg.2012.01.017 PMC3309204

[22] Sachs G, Chang HH, Rabon E, Schackman R, Lewin M, Saccomani G (1976) A nonelectrogenic H+ pump in plasma membranes of hog stomach. J Biol Chem 251:7690–7698 12175

[23] Sanders SW (1996) Pathogenesis and treatment of acid peptic disorders: comparison of proton pump inhibitors with other antiulcer agents. Clin Ther 18:2–34. 10.1016/s0149-2918(96)80175-5 8851451 10.1016/s0149-2918(96)80175-5

[24] Schlingmann KP, Weber S, Peters M, Niemann Nejsum L, Vitzthum H, Klingel K, Kratz M, Haddad E, Ristoff E, Dinour D, Syrrou M, Nielsen S, Sassen M, Waldegger S, Seyberth HW, Konrad M (2002) Hypomagnesemia with secondary hypocalcemia is caused by mutations in TRPM6, a new member of the TRPM gene family. Nat Genet 31:166–170. 10.1038/ng889 12032568 10.1038/ng889

[25] Strand DS, Kim D, Peura DA (2017) 25 years of proton pump inhibitors: a comprehensive review. Gut Liver 11:27–37. 10.5009/gnl15502 27840364 10.5009/gnl15502 PMC5221858

[26] Suksridechacin N, Kulwong P, Chamniansawat S, Thongon N (2020) Effect of prolonged omeprazole administration on segmental intestinal Mg(2+) absorption in male Sprague-Dawley rats. World J Gastroenterol 26:1142–1155. 10.3748/wjg.v26.i11.1142 32231419 10.3748/wjg.v26.i11.1142 PMC7093313

[27] Taylor CA, Hutchens S, Liu C, Jursa T, Shawlot W, Aschner M, Smith DR, Mukhopadhyay S (2019) SLC30A10 transporter in the digestive system regulates brain manganese under basal conditions while brain SLC30A10 protects against neurotoxicity. J Biol Chem 294:1860–1876. 10.1074/jbc.RA118.005628 30559290 10.1074/jbc.RA118.005628 PMC6369308

[28] Thebault S, Alexander RT, Tiel Groenestege WM, Hoenderop JG, Bindels RJ (2009) EGF increases TRPM6 activity and surface expression. J Am Soc Nephrol 20:78–85. 10.1681/ASN.2008030327 19073827 10.1681/ASN.2008030327 PMC2615736

[29] Tholen LE, Latta F, Martens JHA, Hoenderop JGJ, de Baaij JHF (2023) Transcription factor HNF1beta controls a transcriptional network regulating kidney cell structure and tight junction integrity. Am J Physiol Renal Physiol 324:F211–F224. 10.1152/ajprenal.00199.2022 36546837 10.1152/ajprenal.00199.2022

[30] Thongon N, Penguy J, Kulwong S, Khongmueang K, Thongma M (2016) Omeprazole suppressed plasma magnesium level and duodenal magnesium absorption in male Sprague-Dawley rats. Pflugers Arch 468:1809–1821. 10.1007/s00424-016-1905-7 27866273 10.1007/s00424-016-1905-7

[31] Topala CN, Groenestege WT, Thebault S, van den Berg D, Nilius B, Hoenderop JG, Bindels RJ (2007) Molecular determinants of permeation through the cation channel TRPM6. Cell Calcium 41:513–523. 10.1016/j.ceca.2006.10.003 17098283 10.1016/j.ceca.2006.10.003

[32] Tuschl K, Clayton PT, Gospe SM Jr, Gulab S, Ibrahim S, Singhi P, Aulakh R, Ribeiro RT, Barsottini OG, Zaki MS, Del Rosario ML, Dyack S, Price V, Rideout A, Gordon K, Wevers RA, Chong WK, Mills PB (2012) Syndrome of hepatic cirrhosis, dystonia, polycythemia, and hypermanganesemia caused by mutations in SLC30A10, a manganese transporter in man. Am J Hum Genet 90:457–466. 10.1016/j.ajhg.2012.01.018 22341972 10.1016/j.ajhg.2012.01.018 PMC3309187

[33] Tuschl K, Mills PB, Parsons H, Malone M, Fowler D, Bitner-Glindzicz M, Clayton PT (2008) Hepatic cirrhosis, dystonia, polycythaemia and hypermanganesaemia–a new metabolic disorder. J Inherit Metab Dis 31:151–163. 10.1007/s10545-008-0813-1 18392750 10.1007/s10545-008-0813-1

[34] van Angelen AA, San-Cristobal P, Pulskens WP, Hoenderop JG, Bindels RJ (2013) The impact of dietary magnesium restriction on magnesiotropic and calciotropic genes. Nephrol Dial Transplant 28:2983–2993. 10.1093/ndt/gft358 24092847 10.1093/ndt/gft358

[35] Walder RY, Landau D, Meyer P, Shalev H, Tsolia M, Borochowitz Z, Boettger MB, Beck GE, Englehardt RK, Carmi R, Sheffield VC (2002) Mutation of TRPM6 causes familial hypomagnesemia with secondary hypocalcemia. Nat Genet 31:171–174. 10.1038/ng901 12032570 10.1038/ng901

[36] Walder RY, Yang B, Stokes JB, Kirby PA, Cao X, Shi P, Searby CC, Husted RF, Sheffield VC (2009) Mice defective in Trpm6 show embryonic mortality and neural tube defects. Hum Mol Genet 18:4367–4375. 10.1093/hmg/ddp392 19692351 10.1093/hmg/ddp392 PMC2766295

[37] Zogzas CE, Aschner M, Mukhopadhyay S (2016) Structural elements in the transmembrane and cytoplasmic domains of the metal transporter SLC30A10 are required for its manganese efflux activity. J Biol Chem 291:15940–15957. 10.1074/jbc.M116.726935 27307044 10.1074/jbc.M116.726935 PMC4965547

