# Supplementary material for: Characterization of intestine-specific TRPM6 knockout C57BL/6 J mice: effects of short-term omeprazole treatment
Source: Pflugers Arch. 2024 Sep 13;477(1):99–109. doi: 10.1007/s00424-024-03017-9 (PMC11711252; doi:10.1007/s00424-024-03017-9)
Supplement: Supplementary file 1 — Supplementary file1 Supplementary Table S1. List of differentially expressed genes in distal colon of Vill1-TRPM6-/- and TRPM6fl/fl mice. Adjusted p < 0.1. Supplementary Figure S1. Metabolic parameters of Vill1-TRPM6-/- and TRPM6fl/fl mice. (a-d) Bar graphs depicting 24-hour feed intake (a), 24-hour water intake (b), 24-hour urine production (c), 24-hour faeces production (d). Values are presented as mean ± SEM (n=12) and statistical significance (p < 0.05) was determined with one-tailed unpaired T-test. (e-f) Uncropped immunoblots of TRPM6 (e) and B-actin (f), both pointed by red arrows, in the proximal colon. Supplementary Figure S2. Quality control of RNA-sequencing analysis. (a) Principal component analysis (PCA) based on normalized counts of six independent RNA samples from the distal colon of three TRPM6fl/fl (WT1-3) and three Vill1-TRPM6-/- (KO1-3) mice generated with DESeq2 R-package. (b) Heatmap of Pearson correlation clustering of the six samples based on variance stabilizing transformed counts generated with DESeq2 R-package. (c) Heatmap showing pairwise correlations of samples based on distribution of sequence reads. (d-e) Gene Ontology (GO) term enrichment analysis (d) and functional annotation (e) of differentially expressed genes. Supplementary Figure S3. Functionality of SLC30A10. Mn2+ levels in serum (a), 24-hour urine (b), and 24-hour feces corrected to 24-hr food intake (c) from TRPM6fl/fl and Vill1-TRPM6-/- mice. (d-f) Zn2+ levels in serum (d), 24-hour urine (e), and 24-hour feces corrected to 24-hour food intake (f). 25Mg2+ uptake by HEK293 cells overexpressing mock, SLC30A10 WT, SLC30A10 D248A, and SLC30A10 (g). Values are presented as mean ± SEM (a-f: n=12; g: N=3 independent experiments). Significant differences (p < 0.05) were determined with one-tailed unpaired T-test (a-f) and One-way ANOVA followed by Tukey post-hoc test (g). * p < 0.05, † p < 0.01. (DOCX 1287 KB) [file 424_2024_3017_MOESM1_ESM.docx]

**TITLE PAGE**

**Characterization of intestine specific TRPM6 knockout C57BL/6J mice: effects of short-term omeprazole treatment**

Anastasia Adella^1^, Lisanne M.M. Gommers^1^, Caro Bos^1^, Pieter A. Leermakers^1^,

Jeroen H.F. de Baaij^1^, Joost G.J. Hoenderop^1, #^

Supplementary files

^1^ Department of Medical BioSciences, Radboud university medical center, Nijmegen, The Netherlands

^#^ Corresponding author:

Joost G.J. Hoenderop

Department of Medical BioSciences, Radboudumc

P.O. Box 9101, 6500HB, Nijmegen, The Netherlands

Phone: +31-24 361 0580, Email: Joost.Hoenderop@radboudumc.nl

Supplementary table 1

| Gene symbol | Gene name | Adjusted p-value | Log2 fold change |
| --- | --- | --- | --- |
| Trpm6 | transient receptor potential cation channel, subfamily M, member 6 | 8.6313E-24 | -1.6053496 |
| Mt1 | metallothionein 1 | 2.6673E-07 | 0.95032173 |
| Ms4a12 | membrane-spanning 4-domains, subfamily A, member 12 | 0.00391354 | -0.7584777 |
| Acer1 | alkaline ceramidase 1 | 0.00546226 | 0.62819588 |
| Wsb1 | WD repeat and SOCS box-containing 1 | 0.00546226 | -0.6286242 |
| Slc30a10 | solute carrier family 30, member 10 | 0.00546226 | -0.9153902 |
| Hnrnpdl | heterogeneous nuclear ribonucleoprotein D-like | 0.01491199 | -0.4740226 |
| Ighv3-6 | immunoglobulin heavy variable 3-6 | 0.01647194 | -1.3709097 |
| Arrdc3 | arrestin domain containing 3 | 0.0205039 | 0.49027083 |
| Igkv5-39 | immunoglobulin kappa variable 5-39 | 0.02109847 | 1.88469232 |
| Gm9926 | predicted gene 9926 | 0.02298421 | -0.5540399 |
| Ighv1-80 | immunoglobulin heavy variable 1-80 | 0.02550265 | 1.77699511 |
| Edn1 | endothelin 1 | 0.02984392 | 0.68908151 |
| Bora | bora, aurora kinase A activator | 0.03821749 | -0.7539564 |
| Eif2s3y | eukaryotic translation initiation factor 2, subunit 3, structural gene Y-linked | 0.04035331 | -0.7199719 |
| Leng8 | leukocyte receptor cluster (LRC) member 8 | 0.04054418 | -0.4456276 |
| Zfhx4 | zinc finger homeodomain 4 | 0.04892243 | 0.59254121 |
| Hoxa11os | homeobox A11, opposite strand | 0.04892243 | -0.5776686 |
| Kyat1 | kynurenine aminotransferase 1 | 0.06084688 | 0.55626829 |
| Myo19 | myosin XIX | 0.06084688 | -0.4678996 |
| Gm15401 | predicted gene 15401 | 0.06084688 | -0.560739 |
| Mis18bp1 | MIS18 binding protein 1 | 0.06170803 | -0.9086328 |
| Fbxo32 | F-box protein 32 | 0.07167506 | 0.4767256 |
| Slc36a1 | solute carrier family 36 (proton/amino acid symporter), member 1 | 0.07167506 | -0.4397189 |
| Ccnl2 | cyclin L2 | 0.07167506 | -0.4783443 |
| Igkv10-94 | immunoglobulin kappa variable 10-94 | 0.0811043 | -2.5843101 |
| Srsf5 | serine/arginine-rich splicing factor 5 | 0.08299813 | -0.4049646 |
| Mir22hg | Mir22 host gene (non-protein coding) | 0.08300559 | -0.6067174 |
| Cip2a | cellular inhibitor of PP2A | 0.09741434 | -0.7527396 |
| Igkv4-70 | immunoglobulin kappa variable 70 | 0.0995118 | -0.0031606 |
| Bcl3 | B-cell lymphoma 3 | 0.0995118 | -0.0102202 |

**Supplementary table 1. List of differentially expressed genes in distal colon of *Vill1*-TRPM6^-/-^ and TRPM6**^fl/fl^ **mice.** Adjusted p < 0.1.

Supplementary figure 1

**
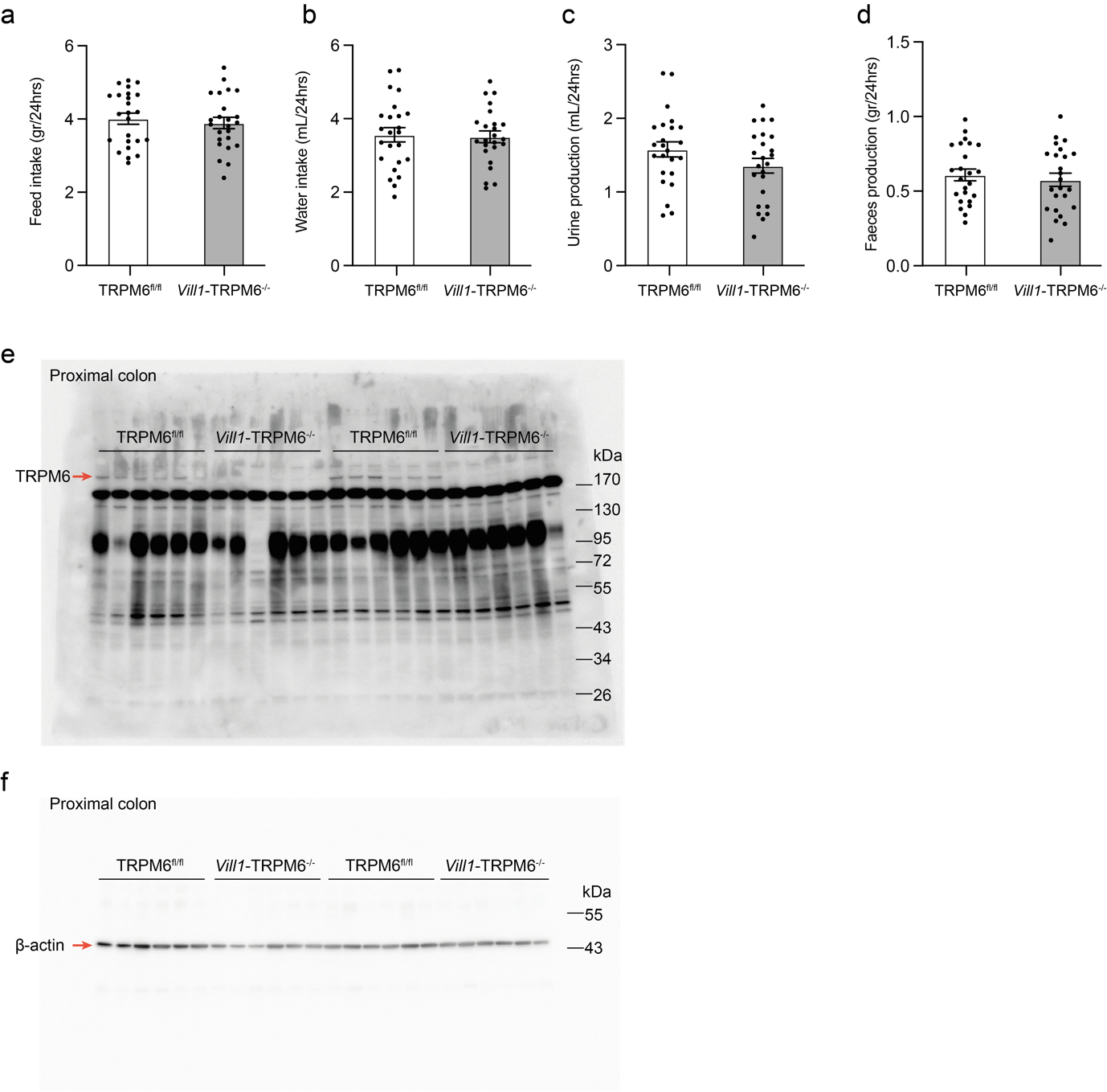
**

**Supplementary figure 1. Metabolic parameters of *Vill1*-TRPM6^-/-^ and TRPM6**^fl/fl^ **mice.** (a-d) Bar graphs depicting 24-hour feed intake (a), 24-hour water intake (b), 24-hour urine production (c), 24-hour faeces production (d). Values are presented as mean ± SEM (n=12) and statistical significance (p < 0.05) was determined with one-tailed unpaired T-test. (e-f) Uncropped immunoblots of TRPM6 (e) and B-actin (f), both pointed by red arrows, in the proximal colon.

Supplementary figure 2


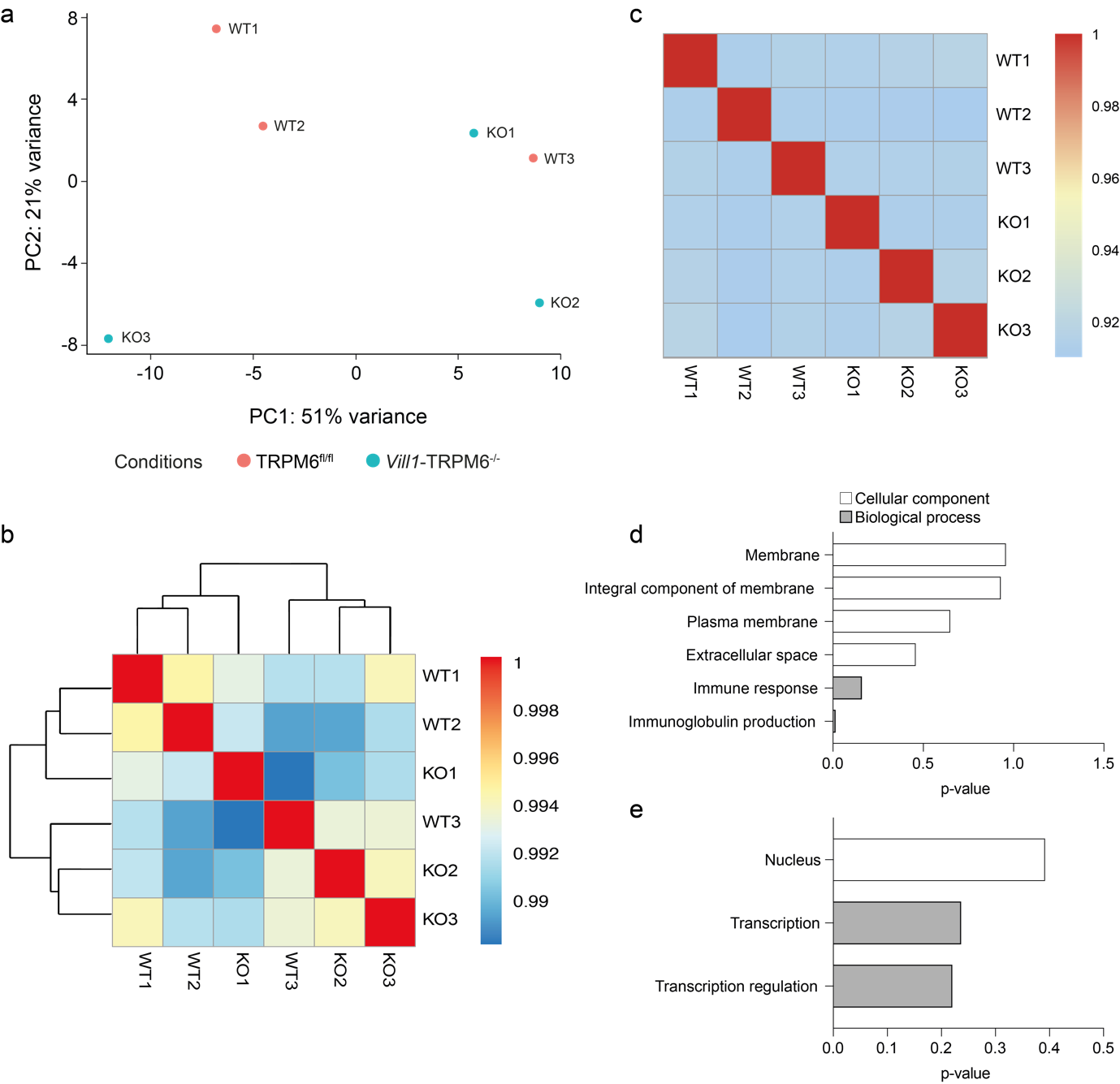


**Supplementary figure 2. Quality control of RNA-sequencing analysis.** (a) Principal component analysis (PCA) based on normalized counts of six independent RNA samples from the distal colon of three TRPM6^fl/fl^ (WT1-3) and three *Vill1*-TRPM6^-/-^ (KO1-3) mice generated with DESeq2 R-package. (b) Heatmap of Pearson correlation clustering of the six samples based on variance stabilizing transformed counts generated with DESeq2 R-package. (c) Heatmap showing pairwise correlations of samples based on distribution of sequence reads. (d-e) Gene Ontology (GO) term enrichment analysis (d) and functional annotation (e) of differentially expressed genes.

Supplementary figure 3


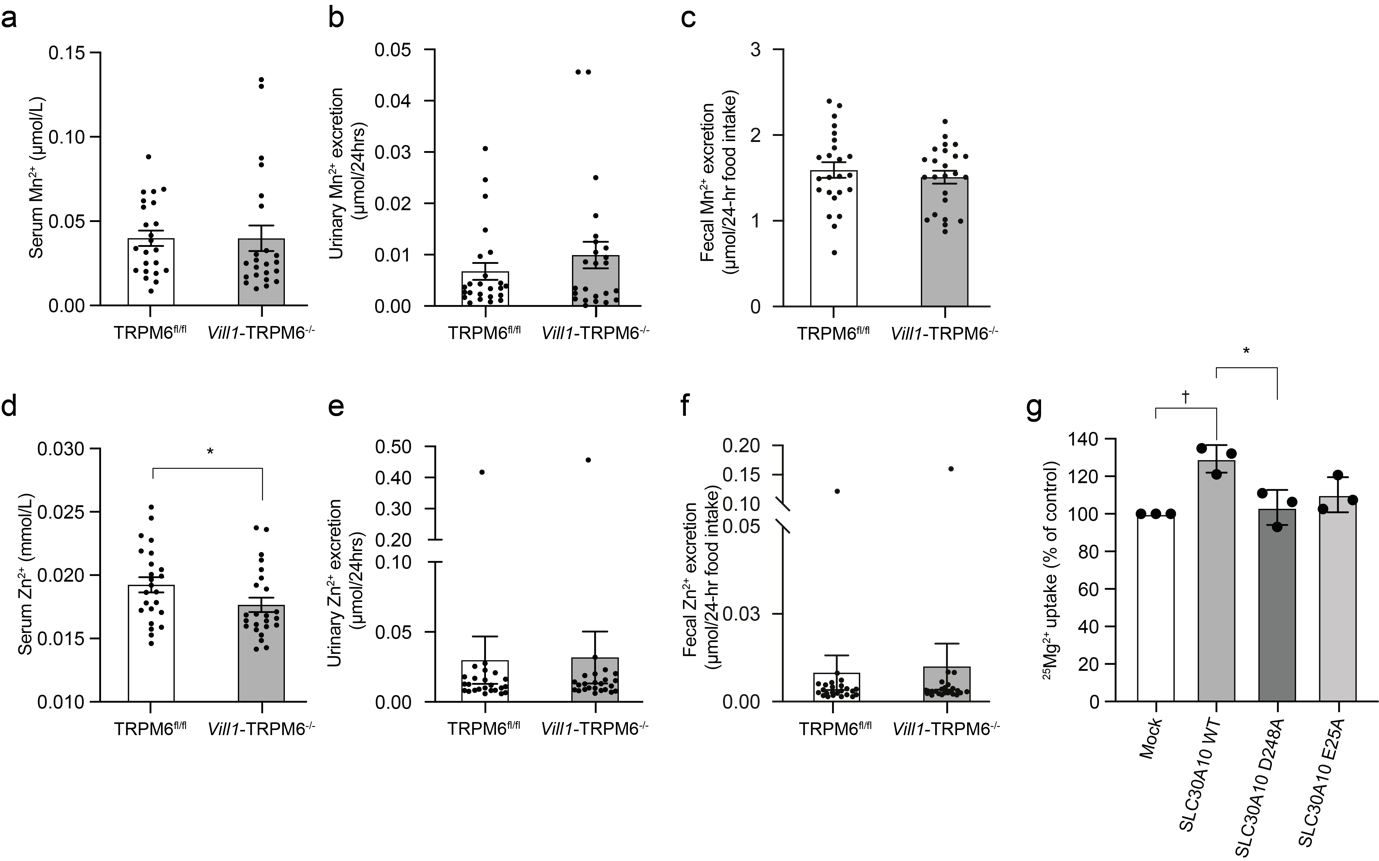


**Supplementary figure 3.** **Functionality of SLC30A10.** Mn^2+^ levels in serum (a), 24-hour urine (b), and 24-hour feces corrected to 24-hr food intake (c) from TRPM6^fl/fl^ and *Vill1*-TRPM6^-/-^ mice. (d-f) Zn^2+^ levels in serum (d), 24-hour urine (e), and 24-hour feces corrected to 24-hour food intake (f). ^25^Mg^2+^ uptake by HEK293 cells overexpressing mock, SLC30A10 WT, SLC30A10 D248A, and SLC30A10 (g). Values are presented as mean ± SEM (a-f: n=12; g: N=3 independent experiments). Significant differences (p < 0.05) were determined with one-tailed unpaired T-test (a-f) and One-way ANOVA followed by Tukey post-hoc test (g). * p < 0.05, † p < 0.01.
